# Effectiveness of venetoclax based therapy in t(11;14) multiple myeloma with extensive extramedullary disease: a case report

**DOI:** 10.1007/s00277-025-06489-6

**Published:** 2025-06-27

**Authors:** Kerstin Brinkert, Bernd Heinrich, Annamaria Brioli, Michael Heuser, Hans Heinrich Kreipe, Florian H. Heidel, Arnold Ganser, Gernot Beutel

**Affiliations:** 1 Department of Hematology, Hemostaseology, Oncology and Stem Cell Transplantation, Hannover Medical School, Carl-Neuberg-Strasse 1, Hannover, 30625 Germany; 2 Department of Gastroenterology, Hepatology, Infectious Diseases and Endocrinology, Hannover Medical School, Hannover, Germany; 3 Institute of Pathology, Hannover Medical School, Hannover, Germany; 4 Cellular Therapy Center (CTC), Hannover Medical School, Hannover, Germany

**Keywords:** Extramedullary myeloma, Venetoclax, t (11;14), Target therapy

## Abstract

Extramedullary disease in patients with newly diagnosed multiple myeloma is rare and, despite the abundance of new therapeutic options, is still associated with a dismal prognosis, classifying as an unmet clinical need. Here, we report the case of a young woman diagnosed with multiple myeloma and extensive extramedullary disease characterised by diffuse lymph node involvement. The patient received multiple chemotherapy regimens proving to be refractory to all of them and ultimately benefited from salvage therapy with Venetoclax combination therapy, with a complete disappearance of the extramedullary manifestations. This case proves the effectiveness of Venetoclax in t(11;14) myeloma with extramedullary involvement.

## Introduction

Multiple myeloma (MM) is a monoclonal plasma cell disorder and is the second most frequent haematological cancer after lymphomas. It primarily affects the bone marrow and is characterised by the presence of monoclonal protein in the serum and/or urine, along with systemic symptoms such as anaemia, bone lesions, hypercalcemia, or renal insufficiency [1]. In rare cases MM can manifest outside the bone marrow, in other organs or in soft tissue, an occurrence named extramedullary MM (EMM). Between 6 and 10% of the patients at diagnosis present with EMM. EMM is associated with the ability of a clone to grow independent of the bone marrow microenvironment, high-risk genetic aberrations and poor overall survival. Despite the remarkable progresses made in the past two decades in the treatment of MM, leading to a significant improvement in overall survival, prognosis of EMM remains dismal, even in the era of new immunotherapies [2, 3]. We present here the case of a patient presenting with a t(11;14) MM and extensive extramedullary involvement, successfully treated with target treatment with Venetoclax.

## Case report

In September 2021, a 21-year-old female was referred from an external hospital to the Department of Haematology, Haemostaseology, Oncology and Stem Cell Transplantation at Hannover Medical School. The patient presented with generalised seizures and the clinical suspicion of hyperviscosity syndrome due to an elevated serum protein (126 g/L) and a very viscous blood, which initially limited detailed laboratory analyses. The patient reported a weight loss of 20 kg, decreasing from 75 kg to 55 kg over a 18-month period, and recurrent episodes of dizziness. Apart from a history of focal nodular hyperplasia of the liver and psoriasis, there were no other pre-existing conditions, and no relevant family history.

**Fig. 1:**
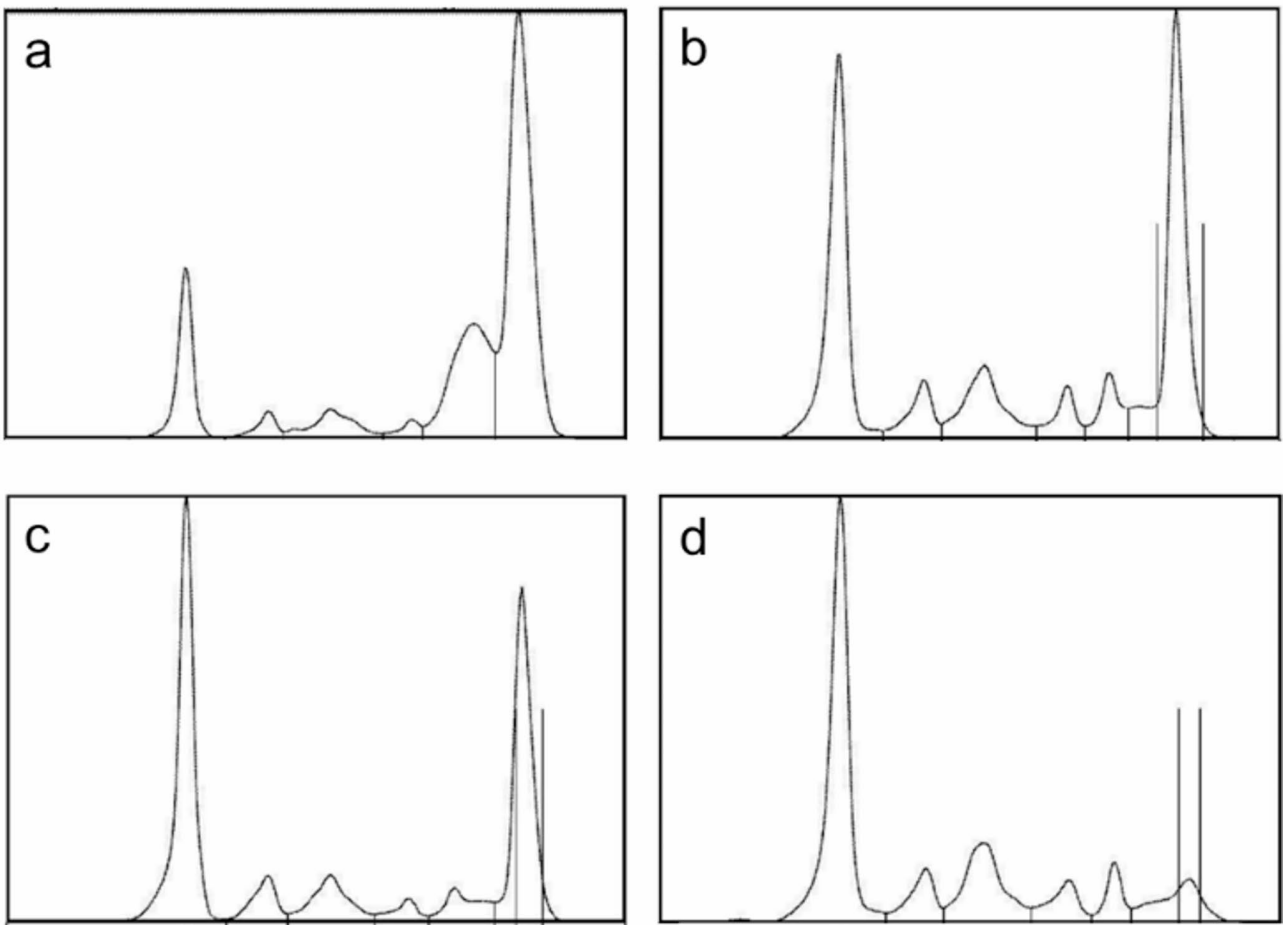
Serum electrophoresis, (**a**) admission, (**b**) after Dara/Bor/Dex, (**c**) after R-Ben-Len, (**d**) after Dara/VEN/Dex

**Fig. 2:**
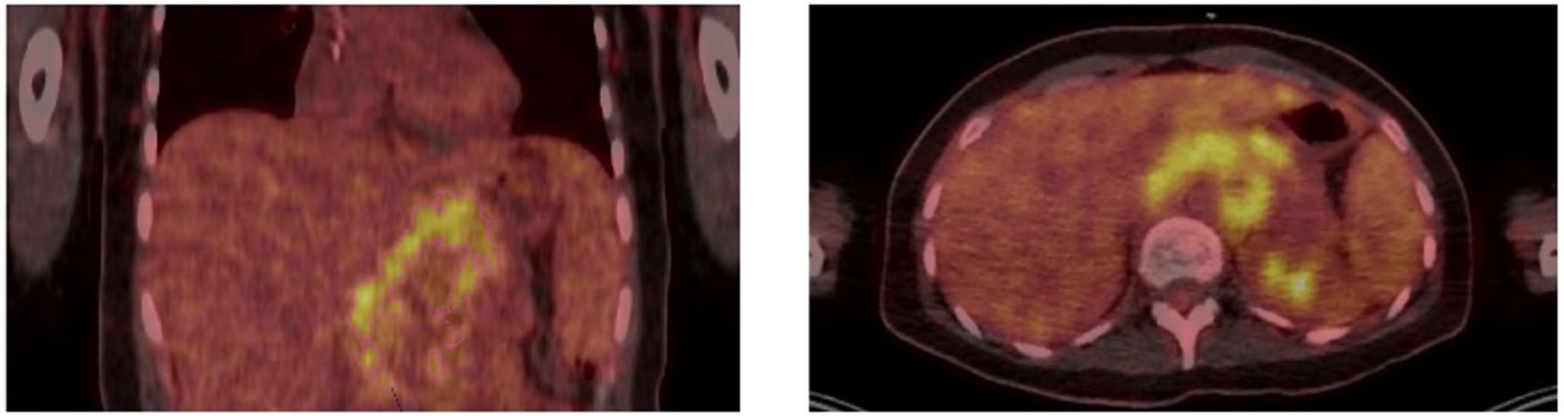
Positron emission tomography, revealing a large mass in the upper abdomen encasing the celiac trunk

Laboratory tests revealed severe anaemia (Hb 7.7 g/dL) with normal white blood and platelet counts, elevated levels of both serum free light chains (kappa 337 mg/L, lambda light chains 73.9 mg/l) an imbalanced kappa/lambda ratio (4.56), elevated γ-globulin fraction (51.6%), a myeloma protein of 46 g/L and ß2 microglobulin of 3.0 mg/L (Fig. 1). While serum albumin (14 g/L) and coagulation factors were decreased, creatinine, calcium, ALT, and LDH levels were within normal limits. The immunofixation showed a monoclonal immunoglobulin IgG-kappa. Positron emission tomography revealed a large mass in the upper abdomen encasing the celiac trunk and enlarged lymph nodes throughout the chest, abdomen, axilla and groin (Fig. 2). No lytic bone lesions were discovered. The bone marrow aspirate showed less than 10% plasma cell infiltration, and the bone marrow biopsy did not demonstrate an excess of monoclonal plasma cells. Cerebrospinal fluid analysis showed normal protein (0.52 g/L) and sugar levels (2mmol/L), mixed pleocytosis and a type 5 monoclonal band. The cell count in the cerebrospinal fluid was 31 cells/ul, which flow cytometry showed as 49% CD3 positive T cells and 16% CD19 positive B cells, which were considered compatible with a reactive picture and not interpreted as a sign of lymphoma. Magnetic resonance imaging of the head, electroencephalogram, echocardiography and lung function test showed no abnormalities.

**Fig. 3:**
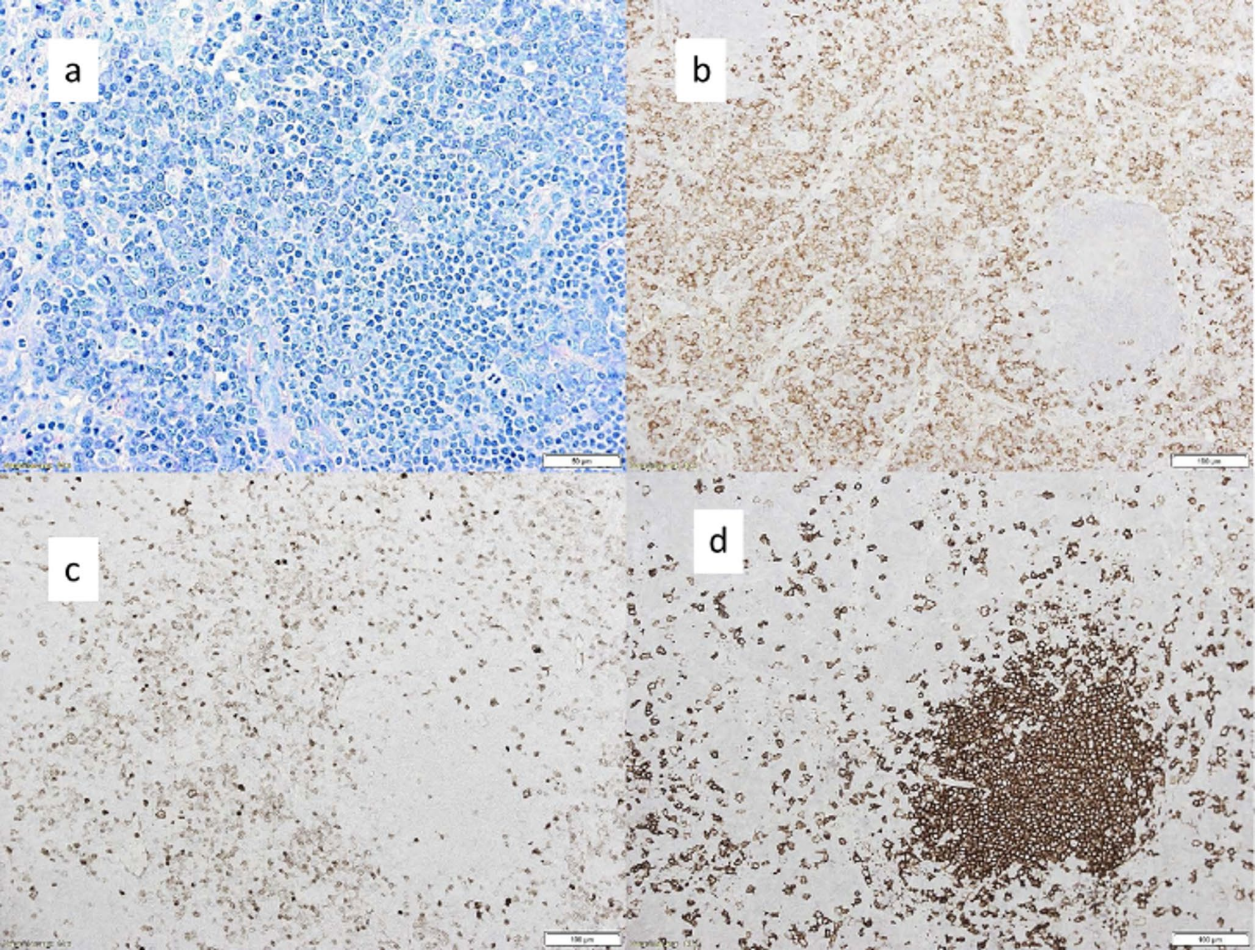
Lymph node histology demonstrated atypical plasma cells in the T-cell area of the lymph node adjacent to residual germinal centres (lower right quadrant) without broadened marginal zone and without Castleman features (**a**). The atypical plasma cell infiltrate revealed kappa light chain restriction (**b**), nuclear cyclin D1 overexpression (**c**) and CD20 negativity (**d**). By contrast the residual germinal centre (lower right quadrant) was negative for kappa light chain (**b**) and cyclin D1 (**c**), but displayed CD20 expression (**d**) (a Giemsa, b-d immunoperoxidase)

The diagnostic workup included repeated tissue biopsies: (1) an exploratory surgery with removal of lymph nodes at the referring hospital, (2) a lymph node biopsy using endobronchial ultrasound, (3) a CT-guided biopsy of a large mass in the upper abdomen which revealed a ganglioneuroma on histology, but did not lead to a definitive diagnosis and (4) last a successful diagnostic laparotomy. During this last diagnostic laparotomy, material from a lymph node near the head of the pancreas and a lymph node in the hepatoduodenal ligament could be successfully obtained. Histological analysis of the lymph nodes initially suggested a Castleman syndrome due to changes in the germinal centres and a monoclonal plasma cell proliferation (Fig. 3). However, the presence of a t (11;14) in 20–30% of the cell nuclei in both analysed lymphnodes argued against a monoclonal plasma cell proliferation in the context of an immunopathological process such as a Castleman disease, so that the final diagnosis of extramedullary plasmacytoma was made. Based on the biopsy-proven extramedullary plasmacytoma, the multiple involved lesions in the positron emission tomography and the anaemia the diagnosis of a MM ISS and R-ISS stage II with extensive extramedullary involvement was made.

As an emergency treatment, the patient underwent repeated plasmaphereses due to clinically suspected hyperviscosity syndrome. Subsequently, the patient was treated with Bortezomib, Dexamethasone, and Daratumumab. Due to insufficient response, Rituximab was added based on the initially suspected diagnosis of Castleman syndrome. However, this treatment approach led to various complications such as bacterial peritonitis, massive edema with pleural effusion, ascites and a prerenal acute kidney injury stage III. Subsequent escalation to Rituximab, Lenalidomide and Bendamustine had to be discontinued due to neutropenia CTCAE grade 4. At this time point the patient had an ECOG of 3 and there was no sign of improvement of the EMM (myeloma protein 21 g/L). Based on data from a phase I study demonstrating the efficacy of Venetoclax, Daratumumab, and Dexamethasone in patients with relapsed or refractory MM, a salvage regimen as described by Bahlis et al. was initiated [4]. 

Therapy was given in 28-day cycles. Venetoclax was given orally 200 mg on day 1–3, 400 mg on day 4–6 and 600 mg thereafter. Daratumumab was given subcutaneously (1800 mg) weekly. Dexamethasone was applied at 40 mg weekly for the first 2 weeks and reduced to 20 mg weekly thereafter.

This approach led to a partial response with a decrease in myeloma protein (Fig. 1), reduced ascites, and clinical improvement, allowing to discharge the patient after several months of inpatient treatment. Unfortunately, the patient was lost to follow up for the following years, so that if the therapy was continued after cycle 2 on an outpatient basis could not be confirmed. The patient was successfully contacted via telephone more than 2 years later, reporting good clinical conditions as well as completing her apprenticeship. A CT scan from June 2024 showed no evidence of tumour. However, the most recent staging imaging from March 2025 showed a renewed cervical, axillary, pectoral, and iliac lymphadenopathy. A repeated lymph node extirpation confirmed the recurrence of the known extramedullary plasmacytoma.

## Discussion

The present report illustrates a successful systemic treatment of MM with t(11;14) and extensive extramedullary involvement. Extramedullary disease in MM is still an unmet clinical need and little prospective data exist for choosing the optimal therapy. Even novel immunotherapies such as CAR-T cells do poorly in this patient’s population. The combination of Teclistamab and Talquetamab as well as CELMoDs seems to be promising but are not approved and available in many countries [2, 3, 5]. In our case, identifying t (11;14) was crucial for diagnosis and determining the most effective treatment approach. The combination therapy containing Venetoclax is a promising alternative treatment option for patients with MM and extramedullary involvement and t (11;14).

Data from a placebo-controlled phase 3 trial (BELLINI) conducted by Kumar et al. showed that adding Venetoclax to a regimen of Bortezomib/Dexamethasone improves progression-free survival in patients with relapsed or refractory MM. In this trial 291 patients were randomly assigned (2:1) to receive Venetoclax or placebo with Bortezomib and Dexamethasone. The overall response rate was 82% in all patients and 90% in patients with t (11;14). Median progression-free survival was 22.4 months with Venetoclax and 11.5 months with placebo. Patients were followed up with a median of 18.7 months. The overall survival was not reached. Although an increase in infections in the Venetoclax group was associated with higher mortality, patients with the t (11;14) mutation experienced a significant survival advantage [6]. Further studies showed that adding Venetoclax improves overall response rate and progression-free survival in patients with relapsed or refractory MM, especially those with t(11;14) [7–11]. Unfortunately the Phase III Trial CANOVA comparing Venetoclax and Dexamethasone vs. Pomalidomide and Dexamethasone did not reach the primary endpoint, and Venetoclax is not approved for the treatment of MM [12]. Nevertheless, the current NCCN Guidelines describe Venetoclax/Dexamethasone with or without Daratumumab or a proteasome inhibitor as an option for patients with MM and a t(11;14) [13]. Target treatment has been successfully used in multiple myeloma, as it is the case also in patients harbouring BRAF mutations, that can be effectively treated with a combination of BRAF and MEK inhibitors [14]. Drawing from this knowledge, we treated our patient according to Bahlis et al. with Venetoclax, Daratumumab and Dexamethasone.

Following refractoriness to multiple lines of treatment with steroids, alkylating agents, proteasome inhibitors, immunomodulatory drugs, anti-CD20, and anti-CD38 antibodies, Venetoclax-based salvage therapy resulted in a clinically significant response and a normalisation of the patient clinical conditions.

Our case highlights the importance of genetic markers in accurately diagnosing and selecting appropriate therapies for patients with MM and extramedullary disease, and reinforce the notion that a genetic guided target therapy could be the way to go in at least a subset of patients with MM. Additionally, it underscores the importance of obtaining multiple biopsies to ensure representative tissue samples, especially in case of uncertain or inconclusive diagnosis.

## Data Availability

No datasets were generated or analysed during the current study.
